# Viscoelastic parameters as discriminators of breast masses: Initial human study results

**DOI:** 10.1371/journal.pone.0205717

**Published:** 2018-10-12

**Authors:** Viksit Kumar, Max Denis, Adriana Gregory, Mahdi Bayat, Mohammad Mehrmohammadi, Robert Fazzio, Mostafa Fatemi, Azra Alizad

**Affiliations:** 1 Department of Physiology and Biomedical Engineering, Mayo Clinic College of Medicine and Science, Rochester, Minnesota, United States of America; 2 Department of Radiology, Mayo Clinic College of Medicine and Science, Rochester, Minnesota, United States of America; University of Montreal, CANADA

## Abstract

Shear wave elastography is emerging as a clinically valuable diagnostic tool to differentiate between benign and malignant breast masses. Elastography techniques assume that soft tissue can be modelled as a purely elastic medium. However, this assumption is often violated as soft tissue exhibits viscoelastic properties. In order to explore the role of viscoelastic parameters in suspicious breast masses, a study was conducted on a group of patients using shear wave dispersion ultrasound vibrometry in the frequency range of 50–400 Hz. A total of 43 female patients with suspicious breast masses were recruited before their scheduled biopsy. Of those, 15 patients did not meet the data selection criteria. Voigt model based shear elasticity showed a significantly (p = 7.88x10^-6^) higher median value for the 13 malignant masses (16.76±13.10 kPa) compared to 15 benign masses (1.40±1.12 kPa). Voigt model based shear viscosity was significantly different (p = 4.13x10^-5^) between malignant (8.22±3.36 Pa-s) and benign masses (2.83±1.47 Pa-s). Moreover, the estimated time constant from the Voigt model, which is dependent on both shear elasticity and viscosity, differed significantly (p = 6.13x10^-5^) between malignant (0.68±0.33 ms) and benign masses (3.05±1.95 ms). Results suggest that besides elasticity, viscosity based parameters like shear viscosity and time constant can also be used to differentiate between malignant and benign breast masses.

## 1. Introduction

Breast cancer is estimated to be the second most common cancer among American women in 2016 [1]. Mechanical properties of soft tissue are related to the physiology and pathophysiology. Any changes in the mechanical properties may indicate the onset or the effects of disease. A broad spectrum of modalities are used in diagnosing breast cancer including ultrasound imaging, shear wave elastography imaging (SWEI), mammography, magnetic resonance imaging (MRI) and magnetic resonance elastography (MRE). Most elastography modalities model the tissue as purely elastic medium and only report the shear modulus [2, 3] of the tissue, whereas in reality the breast tissue or any soft tissue exhibits viscoelastic response. Modeling the soft tissue as purely elastic medium can result in biased estimation of elasticity especially in strongly viscous mediums like breast and liver. A shear wave propagating in viscoelastic medium exhibits frequency dispersion, which means that waves at different frequencies travel at different phase velocities. Ultrasound has been used to non-invasively estimate the tissue viscoelastic parameters. Supersonic shear imaging has been proposed to estimate the elasticity and viscosity of the soft tissue by applying a local inversion algorithm, but has limited real time viability as no *in-vivo* results were presented [4]. Shear wave dispersion ultrasound vibrometry (SDUV) was introduced to quantify the viscoelastic properties of tissue by inducing a shear wave in the range of hundred hertz and then monitoring the shear wave at 1600 frames per second [5, 6]. Liver fibrosis staging has been evaluated using both shear modulus and shear viscosity parameters estimated from SDUV technique [7–10]. Acoustic radiation force imaging (ARFI) has shown the impact of propagating shear wave frequency on fibrosis and steatosis staging [11], and separation of fibrosis staging in non-alcoholic fatty liver disease patients [12]. Time dependent strain analysis (creep deformation at constant stress) of viscoelastic breast tissue under the uniaxial loading assumption can estimate the time constant (retardation time) in the sub-Hertz frequency range [13–16]. Viscoelastic response (VisR) imaging based on the acoustic radiation force calculates the time constant of the Voigt model assuming both the absence and presence of inertial terms [17–21]. VisR has been used to noninvasively monitor renal transplant health [20]. Kinetic acoustic vitreoretinal examination (KAVE) used the Voigt model with an inertial component attached in series to study tissue creep behavior based on an acoustic radiation force push [22]. Lamb wave dispersion analysis has been used to assess the correlation between bladder wall mechanical properties and urodynamic study [23].

Similar to ultrasound, MRI has also been used to characterize the viscoelastic properties of soft tissues. MRI has the advantage of reaching deeper organs and is more suitable for bariatric patients compared to ultrasound. MRE, an MRI based technique for estimating tissue mechanical properties, has been used successfully for determining the stages of liver fibrosis by estimating both the shear modulus and shear viscosity [24–26]. In breast tissue MRE has been used to differentiate between benign and malignant tumors [27] at a frequency of 65 Hz. Shear modulus showed a good separation but shear viscosity was not useful in separating benign and malignant masses [27]. It is noteworthy that MRE is expensive and less universally available. Viscoelastic parameters estimated using wideband MRE have shown sensitivity to alteration of tissue structure in hepatic fibrosis [26].

This study further explores the role of viscoelastic parameters in a wider frequency range for differentiating between benign and malignant breast tumors. To this end, SDUV technique was used to estimate the viscoelastic parameters of *in-vivo* breast masses in the frequency range of 50–400 Hz. SDUV can estimate viscoelastic parameters but has some limitations. The amplitude of the propagating shear wave decays rapidly due to the attenuating media and geometric dispersion which is proportional to the inverse square root of the distance travelled from the source. Especially, malignant breast masses are highly attenuative and shear waves can be observed for only a few milliseconds. Additional factors influencing geometric dispersion are the boundary conditions, the difference in size of scatterer’s and the wavelength of the propagating wave. The medium closer to the push vibrates with higher amplitude and higher particle motion causing the displacement tracking to fail due to decorrelation among the speckles, whereas the region further away from the push sees appreciable geometric dispersion and attenuation. Furthermore, the frequency range of shear waves produced using SDUV is limited to around 400 Hz, as higher frequency shear waves are weak and attenuate quickly. Finally, heterogeneity of tissue introduces error in the shear wave dispersion analysis as the viscoelastic constitutive model assumes a homogenous medium and does not account for the effect of heterogeneity on dispersion. These factors were considered during selection and processing of shear wave data for dispersion analysis. In this study we ignored the effect of geometric dispersion. To the best of the author’s knowledge, this is the first paper demonstrating estimation of viscoelastic parameters using acoustic radiation force in the 50–400 Hz frequency range for shear wave dispersion in breast tissue. Furthermore, this paper shows the correlation between the viscoelastic properties of the suspicious masses with their pathology.

## 2. Material and methods

### 2.1 Theoretical background of viscoelasticity

SDUV technique employs an acoustic radiation force to generate a push inside the soft tissue. The acoustic radiation force is dependent on the attenuation of medium α, acoustic intensity of the ultrasound beam, I, and speed of sound in medium c as described in Eq 1

$$F=\frac{2\alpha Ι}{c}.$$ (1)

The resulting force generates a shear wave which propagates in the medium at a speed c_s_. Assuming a linear isotropic elastic material, the shear wave speed, c_s_, can be written in terms of the shear modulus, μ, of the material and the medium density, ρ, as shown in Eq 2

$$c_{s}=\sqrt{\frac{\mu }{\rho }}.$$ (2)

However, Eq 2 gives a biased estimate of elasticity when the material exhibits viscoelastic properties. To model the frequency dispersion in viscoelastic material, the wave speed has to be estimated as a function of frequency. Different models have been suggested to parametrize the frequency dependent phase velocity, such as the Voigt model, Maxwell model and standard linear solid model. Voigt model has been shown to outperform the Maxwell model for the agar-gelatin phantom and bovine muscles [28]. The effect of inertia is neglected as other studies have shown that the percentage error in the estimated time constant after correction was only 10% [18]. Also due to the complexity of the three dimensional acoustic radiation force push, a correction function had to be derived empirically [18]. The Voigt model consists of a spring and dashpot in parallel, where spring represents the elastic part of the medium and the dashpot represents the viscous part. The propagation of frequency dependent phase velocity, c_s_(ω), in a homogenous Voigt medium is represented by Eq 3

$$c_{s}(\omega )=\sqrt{\frac{2\left(\mu^{2}+\omega^{2}\eta^{2}\right)}{\rho \left(\mu +\sqrt{\mu^{2}+\omega^{2}\eta^{2}}\right)}};$$ (3)

where μ is the shear modulus, ω is the frequency in radians/sec, ρ is the density of the medium and η is the shear viscosity. Both Eqs 2 and 3 assume that the medium is incompressible (Poisson’s ratio = 0.5) in nature and the density of medium stays constant at 1000 Kg/m^3^. Eq 3 can be inversely fitted on the frequency dependent phase velocity data to estimate the shear modulus and the shear viscosity of the medium in which the shear wave is propagating. The time constant of the Voigt model is defined as the ratio of shear viscosity to shear modulus as shown in Eq 4

$$\tau =\frac{\eta }{\mu },$$ (4)

and is representative of the medium in which the shear wave is propagating.

### 2.2 Cohort

The study was approved by the Mayo Clinic Institutional Review Board. Written informed consent was obtained from each patient. Forty-three female patients with suspicious breast masses who were scheduled to undergo biopsy were recruited for the study. The inclusion criteria specified patients over the age of 18, while patients with breast implants, breast abnormality or those who have undergone breast surgery were excluded. Patients were scanned in a supine or lateral oblique position and the location of the suspicious mass and its boundaries were confirmed by a board certified sonographer with 28 years of experience. For each patient at least 4 acquisitions were taken from the suspicious breast mass and at least 2 were taken from a normal breast tissue region. For the recruited patients, SDUV data were collected from the orientation having the biggest lesion cross sectional area, consistent with previous work [2, 29]. For suspicious masses the push location was focused outside the suspicious mass boundary with 2 acquisitions focusing on the left side and 2 on the right side of the suspicious mass boundary. The reasoning behind selection of the push location outside the suspicious mass is provided in section 2.5. The sonographer helped in guiding the location of push in normal tissue.

### 2.3 Experimental setup and data processing

**Fig 1:**
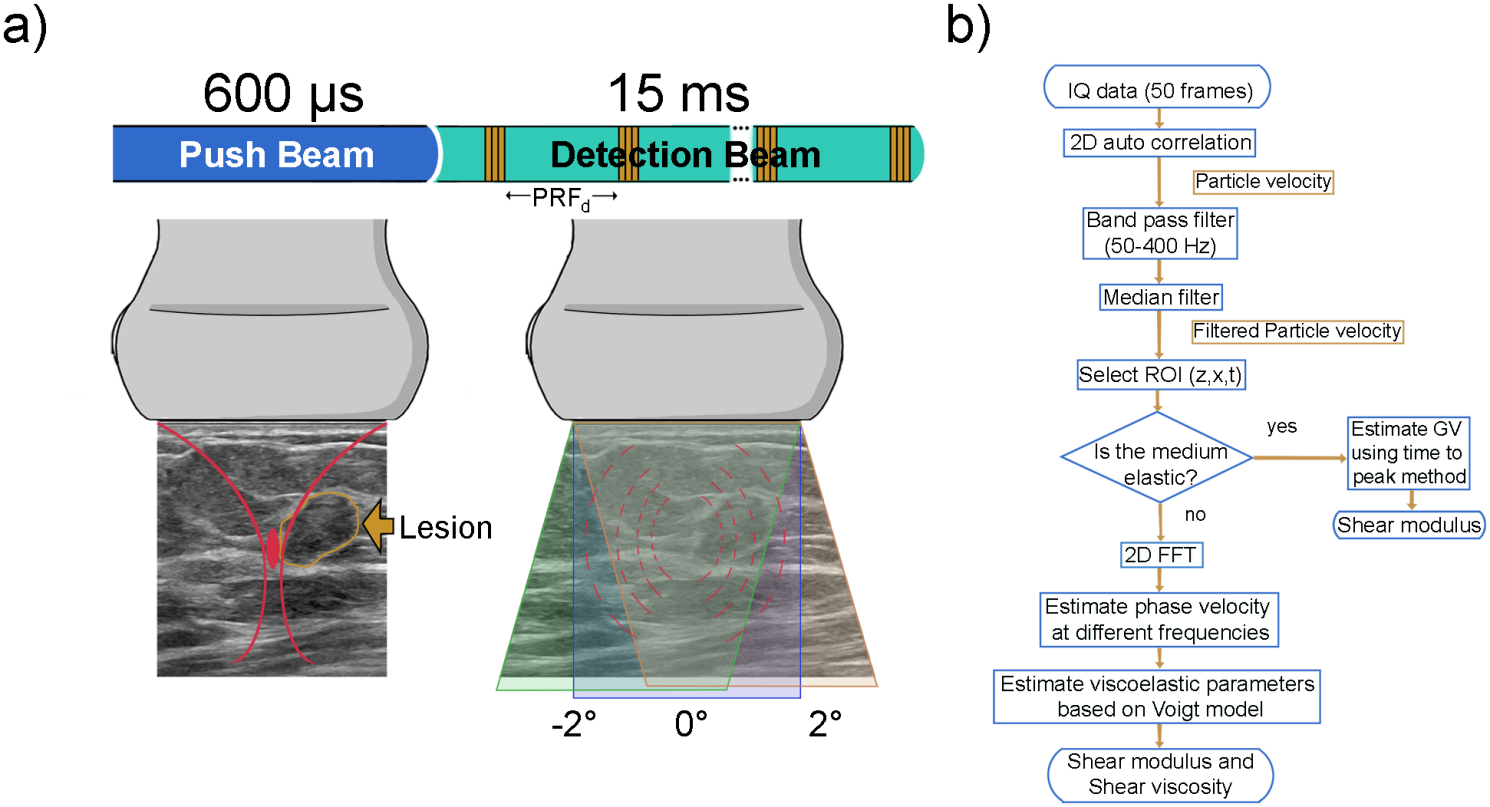
(a) SDUV technique is comprised of a push beam followed by a detection beam. The push beam perturbs the medium at the desired spatial location (illustrated in red) for 600μs. The detection beam is a spatially compounded beam (-2°, 0°, 2°) at a pulse repetition frequency (PRF_d_) of 3.3 kHz for 15 ms and is used to observe the propagating shear wave in the medium. (b) Flowchart summarizing the process of estimating tissue viscoelasticity from the data gathered in the detection phase.

A single transducer can be focused to generate a vibrational motion of the tissue at the desired spatial location followed by compounded plane wave imaging which can track the generated shear wave, as demonstrated in earlier work [6]. A linear array transducer L7-4 (Philips Healthcare, Andover, MA) with a center frequency of 5 MHz was used to create an acoustic radiation force push of duration 600 μs (3 consecutive pushes of 200 μs each). The acoustic radiation force push is followed by 3 angle (-2°, 0°, 2°) compounded plane-wave imaging at a pulse repetition frequency (PRF_d_) of 3.3 kHz for 15 ms using the Verasonics V-1 research system (Verasonics Inc., Redmond, WA) [30]. Fig 1(a) illustrates the push beam and the detection beam associated with SDUV technique. A two dimensional (2D) auto correlation technique [31] was used on the gathered in-phase and quadrature (IQ) data to calculate the particle velocity. Fig 1(b) presents a flowchart summarizing the data processing steps involved in estimation of viscoelastic parameters. The auto correlation technique used a fast axis window length of 2λ (λ = 3.08 μm) and a slow axis window length of 0.9 milliseconds [32]. The particle velocity data was restricted to depths in which the shear wave travelled inside the lesion as determined with the aid of B-mode ultrasound imaging. The particle velocity data was band-pass filtered from 50 Hz to 400 Hz and a median filter of size 3λ × 3λ was applied to reduce the noise spikes due to physiologic and transducer motion [32]. Frequencies higher than 400 Hz were removed as higher shear wave frequencies attenuate faster. Spatio-temporal (xt) maps were generated to display the particle velocity. Particle velocities in the axial direction were averaged over the suspicious mass area. Time to peak velocity estimation method was used to calculate shear wave group velocity in the medium based on a purely elastic medium assumption, as described in Eq 2. To calculate the viscoelastic parameters, based on Eq 3, the method described by Nenadic, et al. [33] was implemented. The frequency dependent phase velocity c_s_(ω) was estimated by calculating a 2D Fourier transform of the particle velocity data for the propagating shear wave and measuring the spatial frequency k(ω) from the location of the peak 2D Fourier transform signal at discrete temporal frequencies. The peak signal at different frequencies was used to calculate the phase velocity at a given frequency. The frequency with the highest signal power was identified as the center frequency of the shear wave. The energy of the 2D-FFT signal was limited to -12dB. Phase velocities higher than 10 m/s were ignored as such high velocities are not feasible. Phase velocity of 10 m/s corresponds to a Young’s modulus of 300 kPa which is much higher than typically observed values of Young’s modulus in breast. The frequency dependent phase velocity is then fitted to the Voigt model based on Eq 3. The problem was modelled as a constrained nonlinear curve-fitting problem in least-squares sense using trust-region-reflective algorithm. Elasticity values were constrained between 0 and 200 kPa whereas viscosity value was constrained between 0 and 100 Pa-s. The shear modulus and shear viscosity were used to estimate the time constant (τ) based on the Voigt model.

All data were processed using MATLAB (The MathWorks Inc., Natick, MA) and MedCalc (MedCalc, Seoul, Republic of Korea). A Mann-Whitney U-test was performed to assess the significant differences in viscoelastic parameters between benign, malignant and normal tissue. P values less than 0.05 were considered to be statistically significant. An IQ data rejection criterion was setup based on the visualization of shear waves inside the lesion. If no shear waves propagating inside the suspicious mass were observed in the xt maps, the IQ data was rejected. This rejection criterion was frequently observed in highly attenuative and smaller sized suspicious breast masses. The shear modulus and shear viscosity estimate for all acquisitions that passed the acceptance criteria were averaged.

### 2.4 Histopathological examination

All patients underwent ultrasound guided core needle biopsy or surgical excision biopsy after the ultrasound research study as part of routine clinical procedure. Five core biopsy samples were obtained for each case by board certified radiologists with more than 15 years of experience using a 14-gauge needle (Achieve biopsy device, CareFusion Corporation, Waukegan, IL). Surgical pathology results matched core biopsy results for the malignant cases. Histopathological results were used as a gold standard to compare the viscoelastic parameters of the suspicious breast masses.

### 2.5 Selection of push location

Since ultrasonic attenuation of normal tissue and pathological tissue can be different [34]. The difference results in a variable force and peak displacement amplitude at the focal region. Different peak displacements at the focal region were reported for different tissue types in ex vivo pigs [10]. Reducing the variability in peak displacement can help in generating pushes which are similar for all suspicious masses, enabling a more controlled method of discriminating those masses. The variability in peak displacement can be reduced by focusing the push in the normal tissue region as the variability in physical properties of normal tissue is low compared to pathological tissue.

Furthermore, the acoustic radiation force push vibrates the medium over a broad range of frequencies and the emanating shear waves have this broadband characteristic. As the shear wave propagates in the medium, its frequency content changes, as higher frequencies are attenuated more than lower frequencies resulting in downshift of the shear wave center frequency. The center frequency at which the medium vibrates at the push location is dependent on the excitation duration and the shear modulus of the medium [35]. The excitation duration can be kept constant experimentally, but shear modulus for pathological tissue and normal tissue is different. Hence, an acoustic radiation force push focused inside the suspicious mass would result in different spectral characteristics of the emanating shear wave when compared to a push focused outside the suspicious mass. If the push is focused in a region of normal tissue, however, the vibrating medium at the push location will have similar mechanical properties, resulting in similar spectral properties of the originating shear wave. The change in acoustic properties of the propagating shear wave would then arise due to the mechanical properties of the medium in which the shear wave is propagating. This change in acoustic properties of the propagating shear wave can be used to differentiate pathological tissues.

For a small sized mass with the push focused inside the mass, interference could arise from the shear wave reflected by the mass boundary resulting in a biased estimation of phase velocity. Moreover, if the small sized mass has high shear modulus, tracking the shear wave inside the mass could be challenging due to the small travel time inside the mass and insufficient sampling rate.

To address the above mentioned concerns, a phantom study was conducted to determine the optimal push location. A CIRS (CIRS, Norfolk, VA) elasticity QA phantom (model 049) with spherical inclusion was used. The inclusion with a radius of 0.492 cm and elasticity of 80 kPa was studied to determine the optimal push location. The phantom does not have a calibrated viscoelastic response but no such calibrated phantom was available commercially. The push was focused both inside and outside the inclusion; the resulting shear waves were studied for optimal push location.

**Fig 2:**
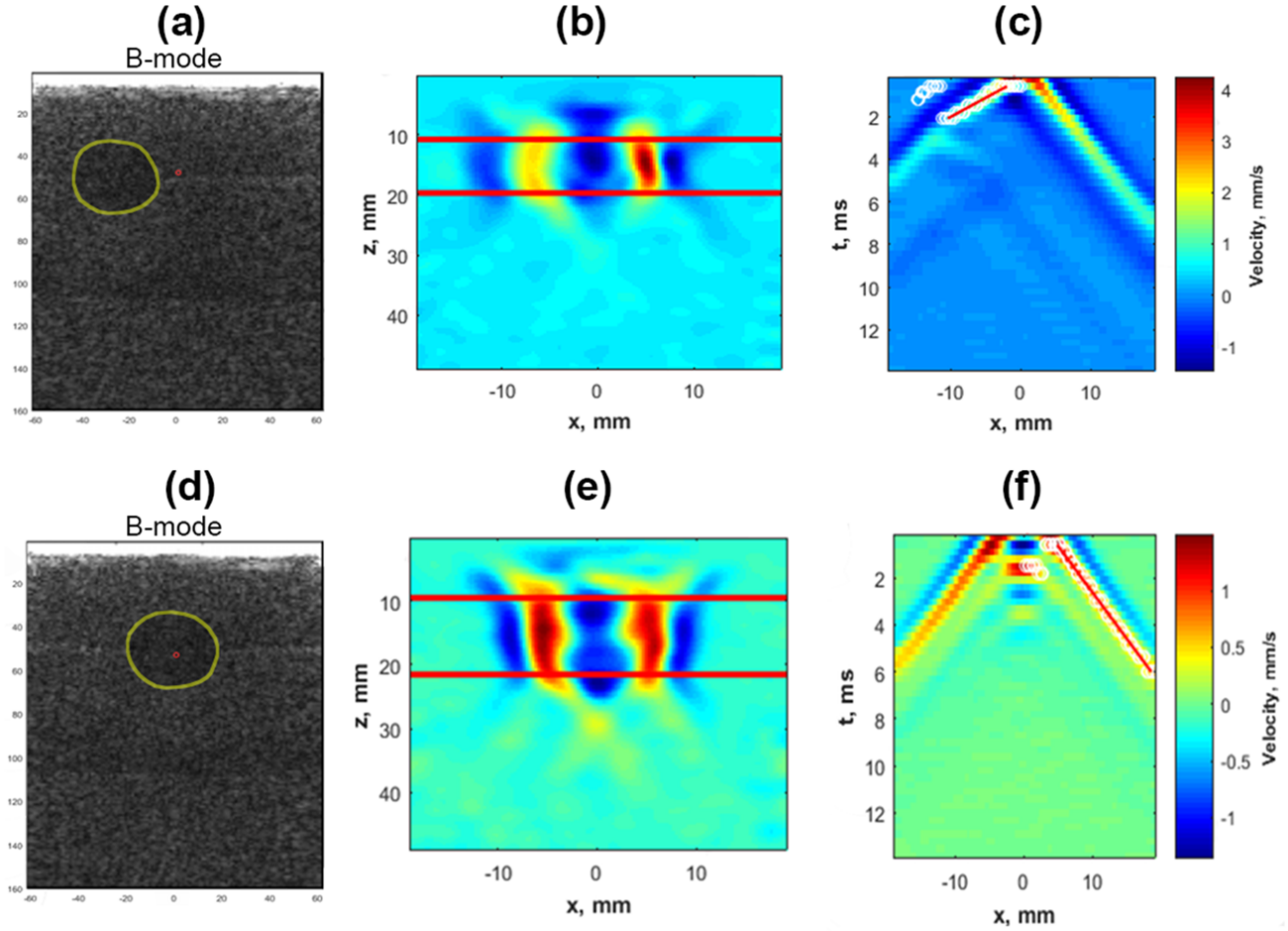
(a) B-mode image of a type IV inclusion (yellow circle) along with push beam location (red dot). (b) Particle velocity maps showing right and attenuated left wing of the shear wave. (c) Spatio-temporal map for the region selected by two horizontal red lines, the estimated group velocity for left shear wave is 5.59 m/s. (d) B-mode image of type IV inclusion along with push beam focused inside the inclusion. (e) Particle velocity map with similar looking left and right wings of shear wave. (f) Spatio-temporal map with estimated group velocity for right shear wave as 2.53 m/s, which corresponds to background material.

Fig 2(a) shows the B-mode image for the elasticity phantom along with push location (shown in red) outside the inclusion. Fig 2(b) illustrates the particle velocity map obtained from displacement tracking of the propagating shear wave. The decrease in particle velocity for the left wing of the shear wave inside the inclusion suggests that the inclusion was stiffer than the background. The two horizontal red lines indicate the depth from which the particle velocities were averaged to construct the xt map of breast tissue motion as shown in Fig 2(c). Part of the propagating shear wave was reflected from the inclusion boundary and can be seen in Fig 2(c) as a wave propagating towards the right side of the inclusion around 3 to 5 ms. The wave selection should be limited to the inclusion region, otherwise it could result in a biased estimate of group velocity. Fig 2(d) shows the B-mode image of the same elasticity phantom but the push was focused inside the inclusion. Fig 2(e) shows the particle velocity map for both the left and right wing of the propagating shear wave. Both wings of the shear wave have similar values of particle velocity unlike Fig 2(b). Fig 2(f) shows the xt map for the phantom material. From the xt map it is difficult to segregate the part of the shear wave which is inside the inclusion from the background. A time-to-peak method for group velocity estimates the speed inside inclusion as 2.53 m/s, which pegs the elasticity estimate around 18.75 kPa by assuming a density of 1000 Kg/m^3^. The estimated elasticity value is closer to background material (25 kPa) than the inclusion (80 kPa), implying that the shear wave was propagating outside the inclusion in the background material rather than inside the inclusion.

Elasticity measurements close to the push are not accurate as the tissue close to the push location vibrates at high amplitude, resulting in poor estimation of particle velocity due to decorrelation in displacement tracking. Hence, if the push is centered inside the suspicious mass, there would be some region inside the suspicious mass for which the elasticity could not be estimated accurately. The diameter of the inclusion used in the phantom study was 1 cm, therefore any mass less than 1 cm with the push focused inside the mass would face the same challenges. For large masses with sufficient size to accommodate both the displacement decorrelation zone and a few milliseconds of shear wave propagation distance, the push could be focused inside. However, when the push location is inside the mass, the vibrating medium at the push location will have acoustic characteristics (center frequency, displacement amplitude) dependent on the mechanical properties of the suspicious mass. In comparison, normal breast tissue has lower variance in viscoelastic properties for different patients. In order to limit the variance in acoustic characteristics of the shear wave, it is recommended to focus the push just outside the suspicious mass boundary in the normal tissue region. The boundaries of suspicious masses can be subjective and B-mode imaging does not depict the true size of the suspicious mass.

### 2.6 Examples of estimation of shear elasticity and viscosity *in-vivo*

**Fig 3:**
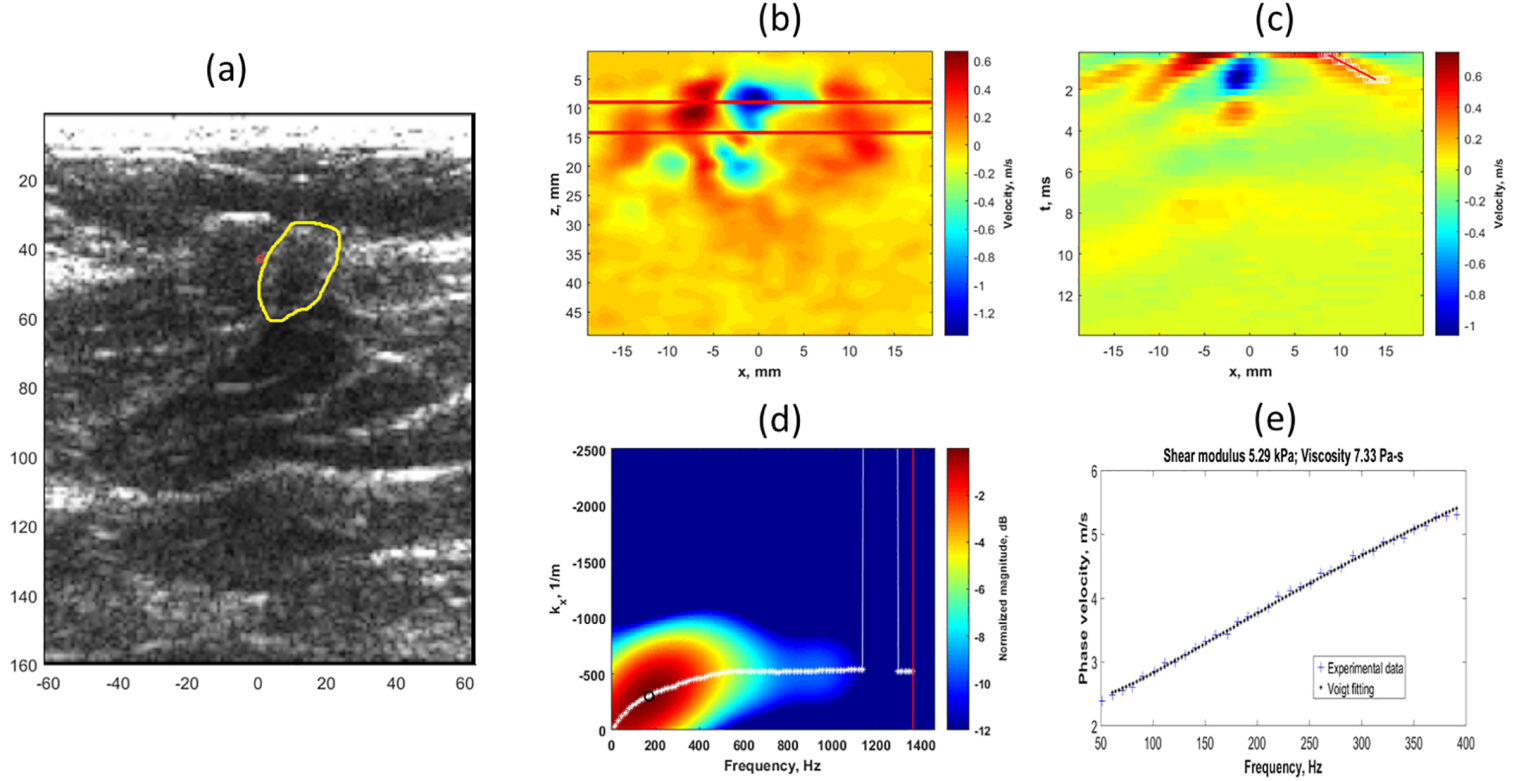
Outline of the process for estimating shear modulus and shear viscosity in a malignant mass. (a) B-mode image of the suspicious map, (b) Particle velocity map showing the left and right wings of shear wave, (c) Xt map showing particle velocity with shear wave marked in red, group velocity was estimated as 4.35 m/s using time to peak method, (d) K-space dispersion map with phase velocity calculated at peak energy for each frequency, (e) Phase velocity experimental data with Voigt model estimation of viscoelastic parameters.

Fig 3 outlines the process for estimating shear modulus and shear viscosity in a malignant mass. Fig 3(a) shows the B-mode image of the suspicious mass (marked in yellow) along with the push location (marked in red). Fig 3(b) presents a single frame showing the particle velocity map. The parallel red lines indicate the region from which the particle velocity data were averaged in the axial direction. Fig 3(c) plots the xt map for axially averaged particle velocity. The group velocity was calculated by tracking the velocity peaks (red line) and determining the slope (c_g_ = dx/dt) of the line. The group velocity for the right wing of the shear wave was 4.35 m/s. Fig 3(d) depicts the K-space map obtained after fast Fourier transform (FFT) of particle velocity data. The center frequency is marked with a black circle. Fig 3(e) plots the phase velocity as a function of frequency. The phase velocity was calculated by taking the peak signal at different frequencies. Shear modulus (5.29 kPa) and shear viscosity (7.33 Pa-s) values were estimated by fitting the phase velocity data to the Voigt model.

## 3. Results

### 3.1 *In-vivo* results

Out of the 43 patients recruited for the pilot study, 28 met the data selection criteria. Thirteen patients had malignant masses (8 ductal carcinoma, 3 mammary carcinoma, 1 lobular carcinoma, 1 metastatic renal cell carcinoma), and 15 had benign masses (6 Fibroadenoma, 3 fibrocystic changes, 3 clustered apocrine cysts, 1 fat necrosis, 1 papilloma, 1 pseudoangiomatous stromal hyperplasia). All suspicious masses had a BIRADS (Breast Imaging Reporting and Data System) score higher than or equal to 3. The suspicious mass size varied from 5.5 to 39 mm in diameter along the greatest dimension, with malignant masses averaging slightly larger than benign (15.56 ± 9.37 mm and 13.19 ± 6.23 mm, respectively). The average age of the cohort was 53.90 ± 14.30 years. Table 1 summarizes the mean and standard deviation values for shear elasticity, viscosity and retardation time constant for each tissue type.

**Table 1:** Mean and standard deviation values of Voigt-based viscoelastic parameters for malignant, benign and normal breast tissue.

Histopathology	Shear elasticity(kPa)	Shear viscosity(Pa-s)	τ (ms)
Malignant (n = 13)	16.76±13.10	8.22±3.36	0.68±0.33
Benign (n = 15)	1.40±1.12	2.83±1.47	3.05±1.95
Normal (n = 28)	1.02±0.97	1.41±0.67	2.55±2.45

**Fig 4:**
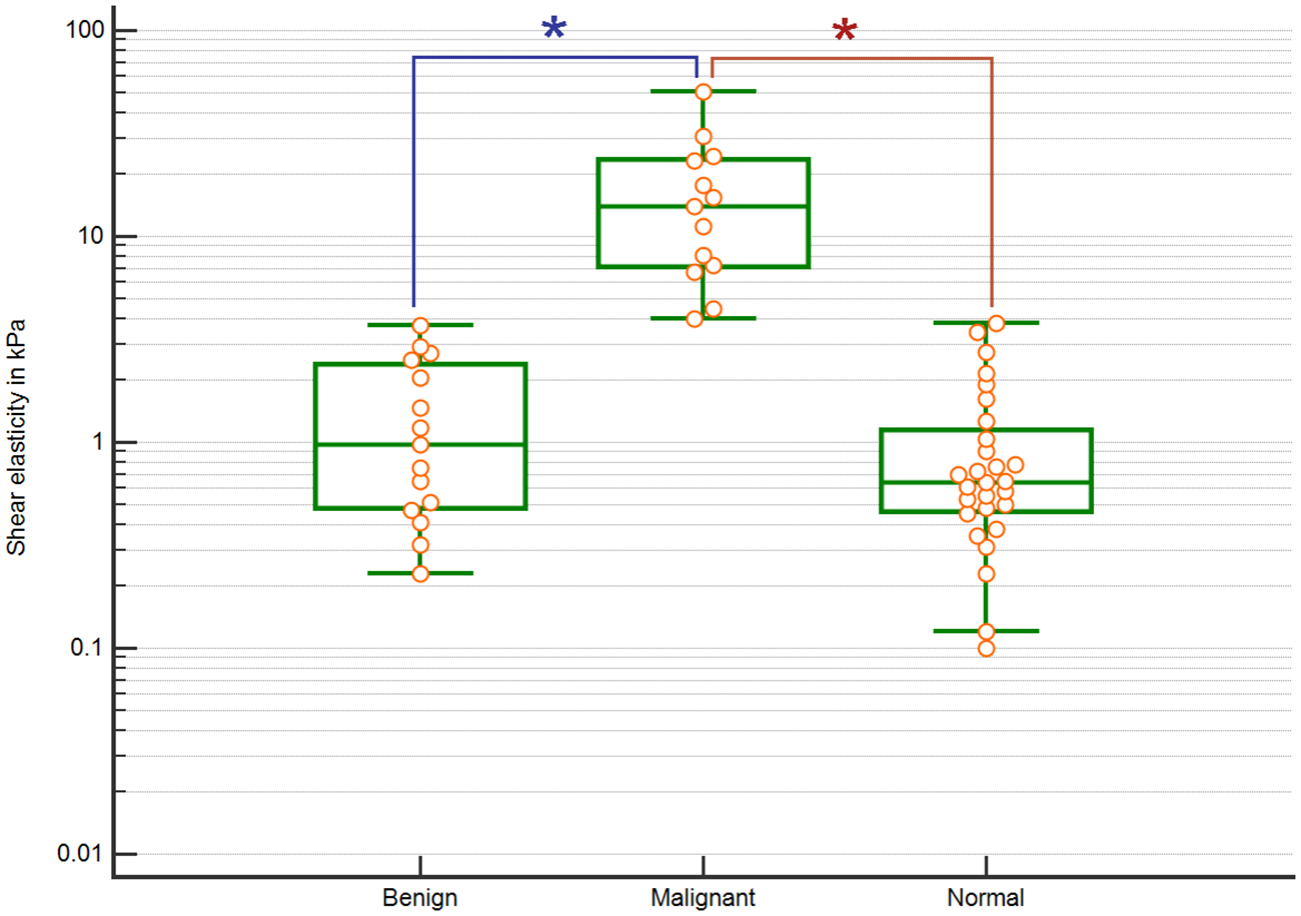
Box plot showing shear modulus distribution for benign masses, malignant masses and normal tissue estimated using a Voigt model with statistically significant differences marked.

Fig 4 shows the box plot for shear modulus of benign, malignant and normal tissue calculated from phase velocity based on the Voigt model. The central box represents the values from 25^th^-75^th^ percentile. The horizontal line inside each box represents the median value. Error bars depict minimum and maximum values. Each datum point (orange circle) is also represented in the box plot. Malignant masses had the highest median value of shear modulus followed by benign masses and normal tissue. The difference between shear modulus for malignant masses and benign masses was statistically significant (p = 7.88*10^−6^). The difference between shear elasticity for malignant masses and normal tissue was also statically significant (p = 3.68*10^−7^). However, shear elasticity cannot significantly differentiate between normal and benign masses. The variance in shear elasticity for malignant masses was much higher compared to benign masses and normal tissue.

**Fig 5:**
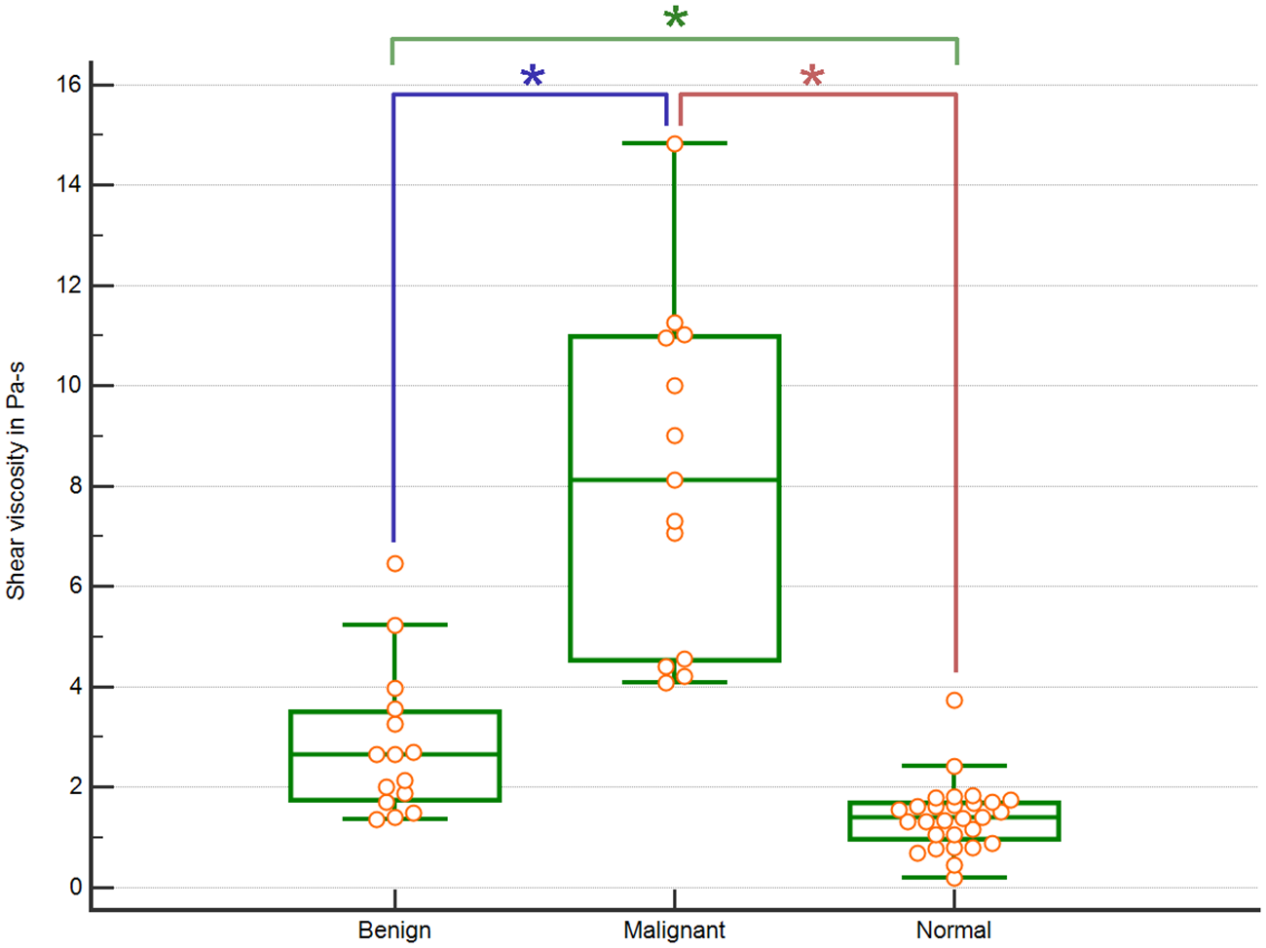
Box plot showing shear viscosity distribution for benign masses, malignant masses and normal tissue estimated using a Voigt model with significantly different pathologies marked.

Fig 5 shows the shear viscosity distribution for benign masses, malignant masses and normal tissue based on the Voigt model. Malignant masses had the highest median value for shear viscosity, followed by benign masses and normal tissue. The variance of shear viscosity in malignant masses was much higher compared to benign masses and normal tissue. Shear viscosity was significantly different between malignant and benign tissue, malignant and normal tissue, and benign and normal (p = 4.13*10–5, 3.67*10–7, and 1.25*10–4, respectively).

**Fig 6:**
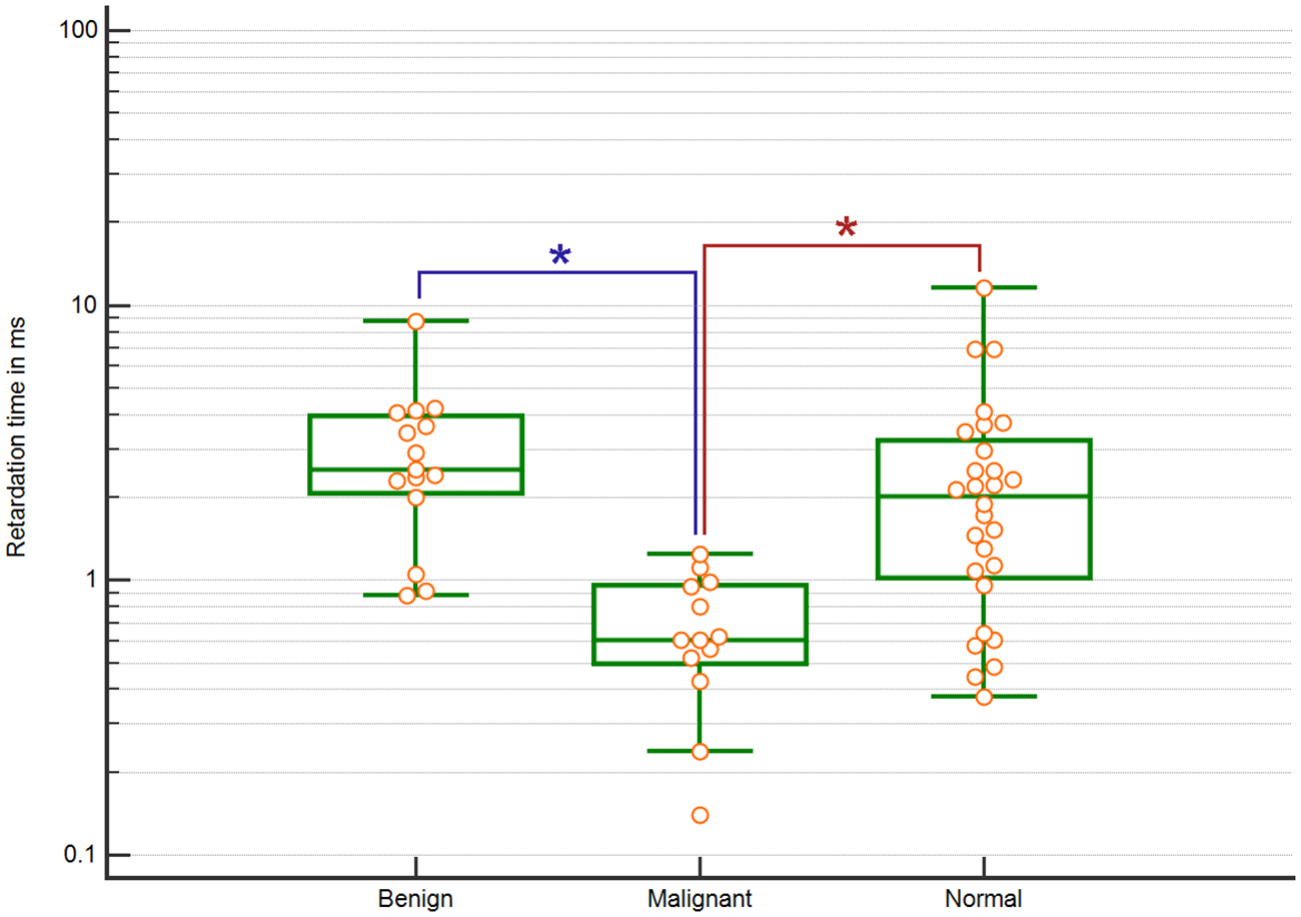
Box plot showing τ distribution for benign masses, malignant masses and normal tissue estimated using a Voigt model with significantly different pathologies marked.

Fig 6 shows the estimated τ distribution in milliseconds for benign masses, malignant masses and normal tissue based on the Voigt model. The median value of τ was lowest in malignant masses and highest in benign masses. The variance in τ of malignant masses was smaller compared to benign masses and normal tissue. Normal tissue had high variance in τ and values overlapped with those of the benign masses; thus, no significant differences were found between the two tissue types. On the other hand, differences in τ values between malignant and benign tissue, and malignant and normal tissue were significant (p = 6.13*10-, and 4.38*10–4, respectively).

## 4. Discussion

This study presents a quantitative analysis of viscoelastic properties exhibited by breast tissue based on the Voigt model. As viscoelastic properties of soft tissue depend on the frequency of excitation and observation time, the viscoelastic properties of *in-vivo* breast tissue reported here are valid only in the frequency range of 50–400 Hz and less than 15 ms of observation time. Other ultrasound based techniques have also reported time constant values of breast tissues in the sub-hertz range [13, 16].

Previous work done in MRE for breast tissue have used a single excitation frequency, thereby limiting the viscoelasticity measurement to that frequency [27]. An impulse excitation, as proposed in previous papers and demonstrated in-vivo in this study, excites a broad range of frequencies, enabling the estimation of viscoelastic parameters from a broader range of frequencies. The limitation of this study is that the frequency range above 400 Hz cannot be observed due to attenuation, which may lead to bias in the estimation of viscoelasticity parameters. In this study all frequencies in the 50–400 Hz range were used to estimate the viscoelastic parameters. The MRI-based method found that malignant masses have higher viscosity than surrounding tissue [27]. It is not feasible to compare the viscosity values estimated from MRE-based study with our study as the excitation frequency in MRE was limited to 65Hz. However, viscosity values from MRE-based study and our study show the same trend with respect to pathology. Similarly, the τ values cannot be compared as the observation time for our study is limited to 15ms and the observation time for MRI-based study was close to 10 min. Although τ cannot be compared quantitatively, the value of τ calculated from Fig 5 of Sinkus et al. [25] reported that the τ for malignant masses was lower than the τ calculated for benign masses, which concurs with our results.

Breast tissue is viscoelastic in nature and should not be modelled as purely elastic medium, since the absence of a viscosity based parameter will lead to bias in estimation of shear elasticity. SDUV enables the estimation of both viscous and elastic parameters of the tissue, leading to better estimation of shear elasticity and additional information related to shear viscosity. The SDUV technique is dependent on the model used to parameterize viscoelastic properties of the medium. A simplistic lumped Voigt model enables easier comparison between benign and malignant masses without the concern of overfitting when compared to higher order models. The shear elasticity for malignant mass was higher compared to benign mass, in accordance with the literature. The Voigt model based shear elasticity for malignant and benign masses was lower than shear wave elastography based elasticity reported in the literature. However, the two shear elasticities should not be compared since the difference stems from the modeling technique, frequency of excitation and overestimation of shear elasticity in shear wave elastography techniques. Furthermore, the effect of dispersion is also ignored in shear wave elastography, resulting in overestimation of shear elasticity, especially in cases with large frequency dispersion [11]. The shear elasticity values from SDUV are in the same range as presented in the literature in the 100 hertz range [27]. The shear elasticity and viscosity values reported here are not an estimation of the ground truth, but an estimation of shear elasticity and viscosity based on the Voigt model.

The shear viscosity values for malignant masses showed a higher spread compared to benign masses or normal tissue implying that some malignant masses might be more viscous than others; this finding is corroborated by other authors [27]. Both shear elasticity and viscosity are parameters obtained by fitting the shear wave phase velocity, hence the two parameters are interrelated to each other and their interaction should be studied together. To study the combined effect of shear elasticity and shear viscosity, it is proposed to use the τ of the Voigt model, defined as the ratio of shear viscosity to elasticity. It is observed that malignant masses have lower τ than normal tissue, whereas benign masses have higher τ than normal tissue as expected from mechano-pathology of pathological tissue.

## 5. Limitations

Based on the phantom study the acoustic radiation push needs to be focused outside the suspicious mass to ensure that the shear wave propagates inside the mass. The identification of region which is outside the suspicious mass can be subjective, since boundaries may not be well-defined and the histological mass size can be larger than its appearance in B-mode imaging [36]. The uncertainty in determining the mass boundary may result in the excited shear wave emanating from the pathological mass rather than the normal tissue, thus having different characteristics and increasing the variability in comparison among the suspicious masses. This limitation can be circumvented by increasing the distance between the push location and the apparent suspicious mass boundary.

Challenges inherent to most elastography techniques, in which the imaging transducer is also used for pushing, is that it is hard to focus near superficial masses while, the push for deeper masses tends to be weak. These limitations apply to SDUV technique as well. A weak push or the presence of a highly attenuative mass makes it harder to track the shear wave.

The selection of window from an xt map plays a crucial role in determining the viscoelastic parameters. If the region selected does not belong to the suspicious mass it can lead to a wrong estimation of viscoelastic parameters, as illustrated in the phantom study. The SDUV technique is open to subjectivity in selection of xt maps and is reliant on proper selection of the shear wave propagating inside the suspicious lesion. In small sized masses the shear wave travels inside the suspicious mass for a limited time, thus only few temporal points are available (dependent on sampling rate), making the estimation of viscoelastic parameters more challenging. Small masses with high stiffness can lead to strain hardening, resulting in small particle displacements with poor quality xt maps. Poor xt maps are also seen in highly attenuative lesions due to the small particle displacements. The above mentioned reasons justify the use of a strict rejection criterion for xt maps, thus rendering the method ineffective for some masses.

Shear wave elastography estimates the speed locally and generates a shear wave speed map. From the shear wave speed map and with the help of B-mode imaging the elasticity of the suspicious mass can be determined. However, creating a viscoelastic map is not feasible with SDUV, as the process would be computationally very intensive. In addition viscoelastic estimation is not a local process as frequency dispersion has to be observed in some spatio-temporal window. Larger spatio-temporal windows provide more reliable estimation of dispersion. On the other hand breast tissue is heterogeneous, thus a bigger window lumps the viscoelasticity parameters in one model. A lumped model with largest possible spatio-temporal window was used in this paper to provide a simplified approach of estimating viscoelastic parameters. In future works, however, the spatio-temporal window can be further split into smaller windows, while ensuring reliability of the estimation process, enabling estimation of viscoelasticity parameters at more spatial locations and a viscoelastic map.

The viscoelastic parameters also depend on the time of observation. Although the time of acquisition is kept constant the time of observation is decided by the limited region of xt map selected for 2D FFT. The time of observation was ignored in this study as the difference in time of observation was expected to be small (few milliseconds) amongst benign and malignant masses.

## 6. Conclusion

Shear elasticity has been shown to differentiate between malignant and benign masses. Our findings demonstrate that viscoelastic parameters like shear viscosity and τ can also be used to differentiate between malignant and benign masses. The high variance of shear viscosity in malignant masses suggests that different breast cancer pathologies might have different viscosity. τ for malignant mass has a very small spread which enables it to be a more efficient and reliable biomarker than shear viscosity. Additional studies should be performed on a larger population to validate these results. Also, phantom experiments demonstrate that it is better to focus the push just outside the suspicious mass in normal tissue to generate similar shear waves for different pathologies.
